# Bacterial Growth on Chitosan-Coated Polypropylene Textile

**DOI:** 10.5402/2012/749694

**Published:** 2012-02-19

**Authors:** D. Erben, V. Hola, J. Jaros, J. Rahel

**Affiliations:** ^1^Department of Physical Electronics, Faculty of Science, Masaryk University, 611 37 Brno, Czech Republic; ^2^Institute for Microbiology, Faculty of Medicine, Masaryk University and St. Anne's Faculty Hospital in Brno, 656 91 Brno, Czech Republic; ^3^Department of Biology, Faculty of Medicine, Masaryk University, 625 00 Brno, Czech Republic; ^4^Centre of Cellular Therapy and Tissue Replacements, 2nd Faculty of Medicine, Charles University, 150 06 Prague, Czech Republic; ^5^Department of Experimental Physics, Comenius University, 842 48 Bratislava, Slovakia

## Abstract

Biofouling is a problem common in all systems where microorganisms and aqueous environment meet. Prevention of biofouling is therefore important in many industrial processes. The aim of this study was to develop a method to evaluate the ability of material coating to inhibit biofilm formation. Chitosan-coated polypropylene nonwoven textile was prepared using dielectric barrier discharge plasma activation. Resistance of the textile to biofouling was then tested. First, the textile was submerged into a growth medium inoculated with green fluorescein protein labelled *Pseudomonas aeruginosa*. After overnight incubation at 33°C, the textile was observed using confocal laser scanning microscopy for bacterial enumeration and biofilm structure characterisation. In the second stage, the textile was used as a filter medium for prefiltered river water, and the pressure development on the in-flow side was measured to quantify the overall level of biofouling. In both cases, nontreated textile samples were used as a control. The results indicate that the chitosan coating exhibits antibacterial properties. The developed method is applicable for the evaluation of the ability to inhibit biofilm formation.

## 1. Introduction

Biofilm formation causes serious problems in areas such as medicine, chemistry, mariculture, and many branches of industry such as food, papermaking, and water treatment industry [1, 2]. Undesirable biofouling is found in filtration systems, air-conditioning systems, medical equipment, food processing facilities, and so forth [3, 4]. Prevention and repair of biofilm damage is very expensive and often low efficient. Biofilms are also increasingly blamed for persistent infectious diseases. Biofil-related diseases include dental caries, cystic fibrosis pneumonia, otitis, infectious kidney stones, and many more [5].

 Biofilms also constitute a niche for microorganisms. The major problem is the extreme resistance of microbes to unfriendly conditions, for example, heat, extreme pH, antibiotics, and disinfectants [5, 6]. Biofilm bacteria can often survive as high as 1000 to 1500 times higher concentrations of disinfectants and antibiotics compared to planktonic cells [7]. Therefore, it is desirable to prevent or minimise biofilm formation at its start. Development of novel materials with functional coating exhibiting high resistance to biofilm formation is a very promising solution.

 Chitosan is a natural polysaccharide processed from chitin obtained mainly from shrimp and crab shells. Its monomers are 2-amino-2-deoxy-D-glucose (glucosamine) and N-acetylglucosamine, so it is a partially N-deacetylated chitin. Chitosan has a huge variety of useful physico-chemical and biological properties such as nontoxicity for human organism, chelating and film-forming ability, and antimicrobial properties [8].

 The antimicrobial activity of chitosan was observed against a wide variety of microorganisms [9, 10]. Inhibitory effects against numerous fungi and bacteria were observed [11, 12]. Chitosan was proposed as an antimicrobial agent for food preservation [11, 13], wound dressing [14], and other purposes.

 The mechanism of antimicrobial activity is not fully understood at present time [15]. Several hypotheses have been proposed. The leakage of intracellular components caused by cell permeabilization due to the interaction between positively charged chitosan and negatively charged cell membranes was observed [16]. Another possible mechanism involves binding of chitosan with DNA, which inhibits transcription [17].

 A major obstacle hampering the chitosan coating onto polymer substrates is their generally poor wettability, which adversely affects the adhesion. Conventional “wet chemistry” methods used for imparting the desired adhesive properties present both health and environmental concerns and increase production cost considerably. The application of nonthermal plasmas is an efficient low-cost alternative solution. The interaction of reactive particles and radiation of short wavelengths with material introduced into plasma can significantly change its properties. A number of different processes such as activation, deposition, crosslinking, and grafting can take place due to introduction of a variety of functional groups and modification of the surface free energy [18]. Recently, we were able to address the problem of chitosan coating adhesion by our genuine atmospheric pressure plasma source—DCSBD (diffuse coplanar surface barrier discharge) [19]. This method was used to coat a thin film of chitosan on the surface of a polypropylene nonwoven textile.

 The aim of this study was to develop a method to evaluate the ability of chitosan coating to inhibit biofilm formation. It is based on the actual evaluation of biofouling level by measuring the pressure increase during filtering of contaminated water with the tested textile sample. The method has shown to be simple to carry out, giving some reasonably reproducible quantitative results of biofilm formation resistance.

 Confocal laser scanning microscopy (CLSM) has been used for further evaluation of the antimicrobial properties of the textile. This allows the observation of the biofilm without significantly damaging it, giving information about structure and cell distribution.

## 2. Materials and Methods

### 2.1. Chitosan Coating Procedure

Using an implementation of the DCSBD plasma system into a continuous textile treater, we are able to quickly prepare large amounts of chitosan-coated PP fabric. The DCSBD used is a modification of the device described elsewhere [20], allowing a treatment of thicker textile if needed. The electrode system consists of 11 pairs of 200 mm long and 2 mm wide silver strip electrodes with mutual distance of 1.5 mm, which are deposited on a panel of 96% Al_2_O_3_ ceramics. The electrical insulation of the electrodes from the inner side is mediated by transformer oil, which is also used for cooling. The electrodes are energized by 14 kHz sinusoidal voltage supplied by a high-voltage generator. The DCSBD electrode system is mounted in a prototype of continuous feed roller. In this case, the plasma generator was operated at 2 W/cm^2^, and the samples were passed through plasma for 5 s.

 A nonwoven spun-bonded PP fabric of 50 g/m^2^ was used (PEGAS NONWOVENS s.r.o.). Immediately after the plasma treatment, the textile was immersed into a chitosan solution and stirred for 1 hour at 60°C. The solution was prepared by dissolving 30 g of chitosan powder (ALDRICH Inc., medium molecular weight, 75–85% deacetylated) into 1500 mL of 0.6% (v/v) acetic acid solution. Afterwards, the textile was left to dry at ambient temperature and then rinsed vigorously in 3 L of demineralized water twice to remove free chitosan, then again dried at ambient temperature. Finally, we have verified the efficiency of the coating procedure, which can be found elsewhere [19].

**Figure 1:**
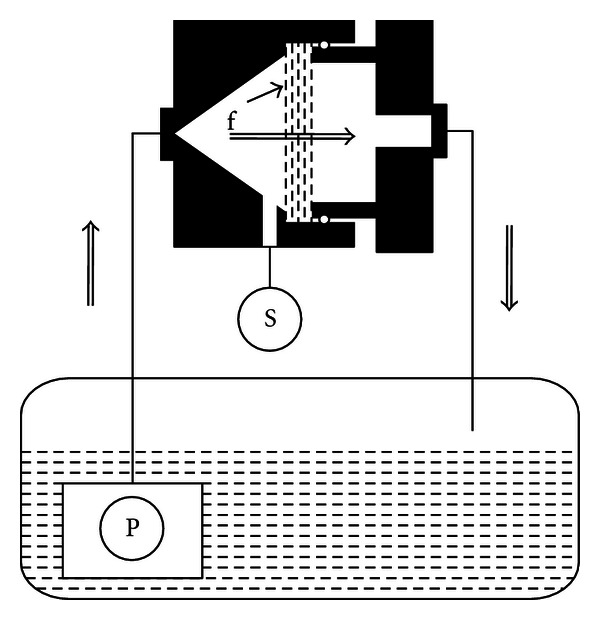
A schematic diagram of the arrangement of the biofilm testing device showing a cross-section of one chamber. (S) pressure transducer, (P) centrifugal pump, (f) filter samples. Double arrows show the direction of the flow.

The biofouling testing device consisted of polyethylene (PE) water tank with two centrifugal pumps drawing water through PE tubing simultaneously into two cylindrical filtration chambers with samples, from where the water returned through PE tubing to the tank. The chambers were made of two parts machined from acetal copolymer ERTACETAL C (Quadrant AG). By screwing them together, the circular filter samples could be fixed in tightly (Figure 1). The chambers were fixed to a chemical support stand by clamps in an invariable height. Pressure transducers TSA (Gefran S.p.A, 0–25 kPa) were used to measure a relative pressure of water on the inflow side of both chambers. Centrifugal pumps Micra (SICCE S.p.A., up to 400 L/h, 0.6 m H_2_O pressure head) were used.

The connection between the two parts of the chambers was sealed with a silicone tension ring, all other connections were sealed with PTFE sealing tape. The connections of separate parts were of brass-or nickel-coated steel, and the pressure sensors were of stainless steel. The signals from the pressure transducers (converting pressure to electric current) were digitalized in a 8 bit A–D converter, transferring the data to a PC. A computer programme was written for logging of the values in required time intervals.

 The water tank was filled with about 1.75 L of natural surface water (without any sterilisation and/or filtration). Water containing wide range of microbial species was desirable. Therefore it was taken from river Svratka several hundred meters below Brno dam. The water was prefiltered through a piece of untreated PP using sintered glass frit to remove larger suspended solids, thus minimizing nonmicrobial filter fouling. A 1,5 g addition of D-glucose (Natura a.s.) was dissolved to increase the exopolysaccharide production. Five circular pieces of textile of 55 mm diameter were fixed into each chamber. One chamber was used with reference samples (no treatment), the second with chitosan-coated samples. The chambers were connected to the water tank, fixed to the support stand, the pumps were started, and the pressure values were logged in 10-minute intervals. To ensure similar conditions for all experiments, pH values were measured at the beginning of each experiment, and temperature was occasionally measured too. The starting pH values were between 7.45 and 8.03, and the temperature stabilized in about 2 hours between 27.7°C and 29.7°C. The experiments ran from 72 to 117 hours. To verify that the pressure development was not caused by nonbiological fouling or other phenomena, one run with water disinfected by addition of methylene blue was conducted. The pressures during this run remained constant for four days.

 Because of high oscillation of the pressures, the measured functions were fitted with adjacent averaging smoothing calculated from series of 20 adjacent points. The minimal value was calculated as an average of 9 measured values around the minimum. To obtain pressure difference, we subtracted this minimal value from the final value, which was calculated as an average of 9 final values. The final value was chosen because in experiment 3 the pressures reached their maxima in approximately 2 days and then significantly dropped. Since we are interested in long-term stable biofouling, the final value is more important than the temporary maximum.

### 2.2. Confocal Laser Scanning Microscopy

For the preparation of samples for microscopy, chitosan-coated and reference samples of about 2 cm^2^ were cut and put together into 100 mL of sterile TSB medium (15 g medium per L litre of demineralized water; HiMedia). Since chitosan coating could be thermally damaged during sterilisation in an autoclave, the samples were sterilised by 70% (v/v) ethanol solution for 5 minutes [21]. The medium was inoculated with 1 mL of GFP labeled *Pseudomonas aeruginosa* (generously provided by W. Kim, USA) solution with optical density 0.5 of McFarland standard and incubated at 33°C for 22 hours at aerobic conditions with occasional shaking. After that, the samples were shortly rinsed (shaking) in 100 mL of tap water, which was repeated thrice to remove unattached bacteria. Immediately after that, they were observed via Olympus IX81 microscope in CLSM mode, using a 405 nm excitation laser.

## 3. Results

### 3.1. Biofouling Device

Three runs on the biofilm testing device were run, and the results are summarized in Table 1.

**Figure 2:**
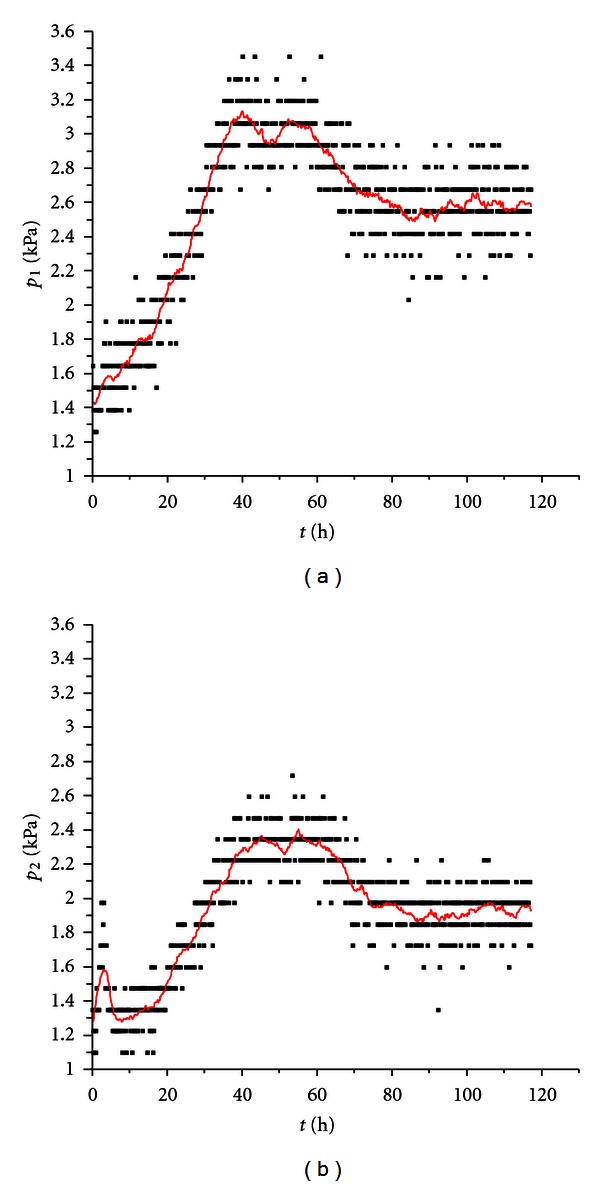
Pressure development in experiment 3. (a) reference, (b) chitosan-coated sample. The discrete character of measured values is caused by digitalization in the 8 bit A–D converter (256 possible values). The fitted lines show adjacent averaging fit as described in the text.

 For experiment 3, graphs of the pressure developments are shown (Figure 2), since it was the longest run and thus shows the largest part of the biofouling process including the final decline of pressure that we assume is corresponding to the death phase and partial release of biofilm, as was shown in our previous studies [22].

 The chitosan-coated filters show lower pressure increase (664 Pa as opposed to 1109 Pa for the reference), suggesting higher biofouling resistance than untreated filters, but the difference was not as high as anticipated, and more work will be required to make certain conclusions possible.

 To summarize, a simple and inexpensive physical method for the evaluation of filter biofouling resistance was developed, and its applicability has been demonstrated. The developed experiment still meets many problems that are currently being addressed. We are now testing the apparatus with a 14 bit A–D converter for finer measurement, and we have also fitted the chambers with flow meters to obtain more information for better characterization of the biofouling process. 

### 3.2. CLSM Imaging

**Figure 3:**
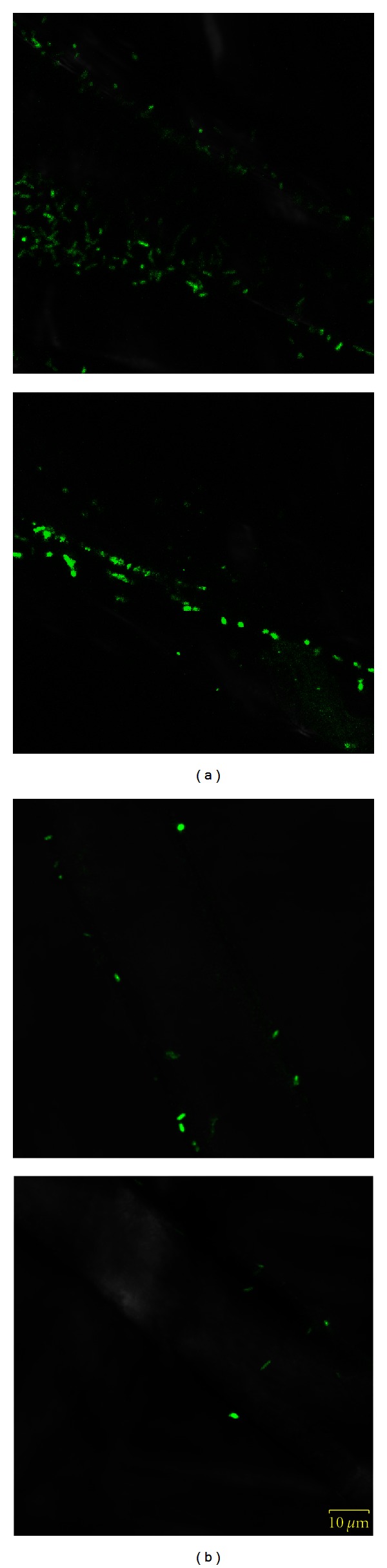
CLSM images of reference (a) and chitosan-coated sample (b). Images were created by merging CLSM and light microscopy photographs. The difference in cell coverage and presence of biofilm matrix is obvious.

As can be seen on the microphotographs (Figure 3), chitosan-coated samples are covered by significantly less bacterial cells compared to the reference, and the bacteria are rather sparsely distributed, while on the reference sample, they form a thicker film. Counting of the cells on 8 randomly chosen fibre sections of both samples showed that chitosan-coated sample is covered by approximately 5 times less cells per length unit of fibre.

## 4. Discussion and Conclusions

Our experiments have shown the validity of the developed tool for quantitative assessment of biofouling resistance. The early results indicate higher biofouling resistance of the chitosan-coated textile and growth and/or adhesion suppression of *Pseudomonas aeruginosa*. However, the presence of bacteria and the measured decrease of filterability on chitosan filters implicate limited use of the chitosan-coated textile in its current form. The effect will probably vary significantly with various bacterial strains, so experiments with other strains might yield some positive results. Possibly, different forms of chitosan and different textile treatment procedures could increase the antimicrobial efficiency. We have already prepared a procedure for binding heavy metal ions to the chitosan coating, which should further increase the biocide properties.

 There are microorganisms resistant to chitosan, such as some fungi having chitosan as a major component of cell walls [23]. Also, it is known that some bacteria are efficient producers of chitosanases—enzymes that attack chitosan by endohydrolysis of beta-1, 4-linkages between D-glucosamine residues in a partly acetylated chitosan [24]. According to the study, the production of chitosanases can be significantly inactivated by the presence of Cu^2+^ ions. The copper ions also have a biocide effect. This might speak for use of chitosan-coated filters with adsorbed Cu as a material having higher antimicrobial effect. We are proceeding to test this hypothesis.

 To summarize, these early results support the hypothesis of bacterial growth/attachment suppression and further research into the application of a thin-film chitosan coating as an antibiofouling agent, which we are currently conducting.

## Figures and Tables

**Table 1:** Pressure differences for three experiments. Δ*p*_1_ is for reference sample, Δ*p*_2_ for chitosan-coated sample. Standard deviations are displayed.

Exp. #	Time/h	Start pH	Δ*p*_1_/Pa	Δ*p*_2_/Pa
1	72	8.03	1547 ± 78	1050 ± 55
2	96	7.92	702 ± 67	554 ± 53
3	117	8.01	1109 ± 70	664 ± 66

## Abbreviations

A–D: Analog-digital
CLSM: Confocal laser scanning microscopy
DCSBD: Diffuse coplanar surface barrier discharge
GFP: Green fluorescent protein
PE: Polyethylene
PP: Polypropylene
PTFE: Polytetrafluoroethylene
TSB: Tryptone soya broth.
